# Increased *MYBL2* expression in aggressive hormone‐sensitive prostate cancer

**DOI:** 10.1002/1878-0261.13314

**Published:** 2022-10-02

**Authors:** Yuki Yoshikawa, Konrad H. Stopsack, Xin Victoria Wang, Yu‐Hui Chen, Ying Z. Mazzu, Foster Burton, Goutam Chakraborty, Sai Harisha Rajanala, Rahim Hirani, Subhiksha Nandakumar, Gwo‐Shu Mary Lee, David Frank, Elai Davicioni, Glenn Liu, Michael A. Carducci, Haruhito Azuma, Philip W. Kantoff, Christopher J. Sweeney

**Affiliations:** ^1^ Department of Medicine Memorial Sloan Kettering Cancer Center New York NY USA; ^2^ Department of Urology Osaka Medical and Pharmaceutical University Japan; ^3^ ECOG‐ACRIN Biostatistics Center Dana‐Farber Cancer Institute Boston MA USA; ^4^ Department of Medical Oncology Dana‐Farber Cancer Institute Boston MA USA; ^5^ Department of Urology Icahn School of Medicine at Mount Sinai New York NY USA; ^6^ Center for Molecular Oncology Memorial Sloan Kettering Cancer Center New York NY USA; ^7^ Decipher Biosciences Inc. San Diego CA USA; ^8^ University of Wisconsin Carbone Cancer Center Madison WI USA; ^9^ Sidney Kimmel Cancer Center Johns Hopkins Medical Institutions Baltimore MD USA

**Keywords:** androgen deprivation therapy, cell cycle, CHAARTED, docetaxel, MYBL2

## Abstract

Loss of the histone demethylase KDM5D (lysine‐specific demethylase 5D) leads to *in vitro* resistance of prostate cancer cells to androgen deprivation therapy (ADT) with and without docetaxel. We aimed to define downstream drivers of the KDM5D effect. Using chromatin immunoprecipitation sequencing (ChIP‐seq) of the LNCaP cell line (androgen‐sensitive human prostate adenocarcinoma) with and without silenced *KDM5D, MYBL2*‐binding sites were analyzed. Associations between *MYBL2* mRNA expression and clinical outcomes were assessed in cohorts of men with localized and metastatic hormone‐sensitive prostate cancer. *In vitro* assays with silencing and overexpression of *MYBL2* and *KDM5D* in androgen receptor (AR)‐positive hormone‐sensitive prostate cancer cell lines, LNCaP and LAPC4, were used to assess their influence on cellular proliferation, apoptosis, and cell cycle distribution, as well as sensitivity to androgen deprivation, docetaxel, and cabazitaxel. We found that silencing *KDM5D* increased histone H3 lysine K4 (H3K4) trimethylation and increased *MYBL2* expression. *KDM5D* and *MYBL2* were negatively correlated with some but not all clinical samples. Higher *MYBL2* expression was associated with a higher rate of relapse in localized disease and poorer overall survival in men with metastatic disease in the CHAARTED trial. Lower *MYBL2* levels enhanced LNCaP and LAPC4 sensitivity to androgen deprivation and taxanes. *In vitro*, modifications of *KDM5D* and *MYBL2* altered cell cycle distribution and apoptosis in a cell line‐specific manner. These results show that the transcription factor *MYBL2* impacts *in vitro* hormone‐sensitive prostate cancer sensitivity to androgen deprivation and taxanes, and lower levels are associated with better clinical outcomes in men with hormone‐sensitive prostate cancer.

## Graphical abstract

Higher levels of the transcription factor MYBL2 increase the aggressiveness and decrease the sensitivity of hormone‐sensitive prostate cancer to androgen deprivation and docetaxel.

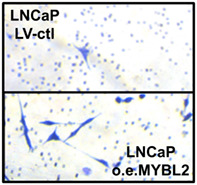

**Abbreviations**
ADT: androgen deprivation therapy
AR: androgen receptor
ATCC: American‐type culture collection
ChIP: chromatin immunoprecipitation
CRPC: castration‐resistant prostate cancer
CSS: charcoal‐stripped serum
ctl: control
DHT: dihydrotestosterone
DOC: docetaxel
FFPE: formalin‐fixed, paraffin‐embedded
HBSS: Hank's balanced salt solution
HR: hazard ratio
INT: iodonitrotetrazolium chloride
LV: lentivirus
mHSPC: metastatic hormone‐sensitive prostate cancer
o.e.: overexpression
OS: overall survival
PrCa: prostate cancer
RIPA: radioimmunoprecipitation assay
SCAN: single channel array normalization
scr: scramble
TCGA: The Cancer Genome Atlas

## Introduction

While some men with metastatic hormone‐sensitive prostate cancer (mHSPC) treated with testosterone suppression (also referred to as androgen deprivation therapy, ADT) alone have a rapid tumor progression and die within months from starting therapy, others will have durable responses lasting more than a decade. Clinical features associated with poorer overall survival (OS) with ADT alone include a higher disease burden and presenting with metastatic disease at the time of initial diagnosis [1, 2, 3]. Notably, men with features suggestive of a rapidly progressive cancer (*de novo* presentation with high‐volume disease) have a marked and consistent improvement in OS with the addition of docetaxel to ADT. By contrast, the addition of an extragonadal androgen synthesis inhibitor (abiraterone) or potent direct androgen receptor (AR) inhibitor (enzalutamide or apalutamide) to ADT imparts clear OS benefits for patients with both low‐ and high‐burden disease [4, 5, 6].

To date, little is known regarding the biological basis for an aggressive course and who benefits from the addition of docetaxel, abiraterone, enzalutamide, or apalutamide to testosterone suppression. Alterations in cell cycle control mechanisms including *RB1* are strongly associated with shorter time to castration resistance and OS, and preliminary data suggest that cell cycle alterations might be enriched in poor‐prognosis high‐volume disease [7, 8, 9].

In prior preclinical work [10] we found prostate cancer cells with low KDM5D were less sensitive to docetaxel and that this was more apparent in the presence of androgens. This effect was partially reversed by AR inhibition with enzalutamide. By contrast, silencing KDM5D in the AR‐negative cell line DU145 that has high KDM5D did not alter sensitivity to docetaxel. Together, this suggests an interaction between AR activity and KDM5D in prostate cancer biology. In human clinical specimens, low *KDM5D* expression was associated with a higher risk of metastasis after prostatectomy and progression to castration resistance as well as shorter OS. One potential cause of low *KDM5D* expression in advanced prostate cancer is complete copy number loss given its localization on the Y chromosome.

To identify potential downstream mediators of the effects of KDM5D, we deployed chromatin immunoprecipitation (ChIP)‐sequencing and discovered that KDM5D physically interacts with AR in the nucleus and regulates its transcriptional activity by demethylating H3K4me3‐active transcriptional marks [10]. Thus, loss of KDM5D leads to increased expression of AR‐regulated genes. RNA‐sequencing analyses and functional preclinical work also suggested that KDM5D loss leads to acceleration of the cell cycle with mitotic entry and increased DNA‐replication stress [11]. We also showed that loss of KDM5D, consistent with its demethylating function, caused increased levels of H3K4me3 and H3K4me2 at additional KDM5D binding sites in LNCaP cells. Subsequent motif analyses suggested KDM5D was a coregulator of multiple transcriptional factors of the cell cycle, such as the E2F family and notably MYBL2.

In this study, we demonstrate that MYBL2, an MYB transcription factor family member, is one of the key mediators of KMD5D's biological effects and that *MYBL2* levels impact resistance to androgen deprivation and docetaxel. It has been previously reported that higher levels of MYBL2 are associated with poor prognosis in both hematological and solid tumors[12, 13, 14, 15] including prostate cancer [16]. In addition, it has been shown that MYBL2 increases cell survival, cell differentiation, and cell cycle progression. MYBL2 also interacts with FOXM1, another transcription factor with a key role in cell cycle regulation [17]. We hypothesized that MYBL2 is one of the important regulators of the cell cycle and proliferation in hormone‐sensitive prostate cancer and that its upregulation upon loss of KDM5D is one of the mediators of progression to relapse and therapy resistance.

## Materials and methods

### Cell lines and proliferation assay

The prostate cancer cell lines LNCaP, 22RV1, and DU145 were obtained from the American Type Culture Collection (ATCC). The LNCaP‐C42 cell line was obtained from ViroMed Laboratories. The E006AA cell line was provided by John T. Isaacs (Johns Hopkins, Bltimore, MD, USA), and the LAPC4 cell line was provided by Charles Sawyers (Memorial Sloan Kettering). Cells were maintained in a medium (RPMI 1640) containing 10% FBS (LNCaP, 22RV1, DU145, LNCaP‐C42, LAPC4, E006AA) or 10% charcoal‐stripped serum (LNCaP and LAPC4) supplemented with 2 mm of L‐glutamine at 37 °C in 5% CO_2_. The viability of cells treated in each experiment was determined using the Cell Titer‐Glo Luminescent Assay (Promega, Madison, WI, USA) and incubating the cells in a 96‐well format for 10 min with a luminescent reagent in a 1 : 1 medium according to the manufacturer's protocol.

### ChIP‐sequencing

Cells (7.5 × 10^6^) at room temperature were cross‐linked for 10 min with 1% paraformaldehyde and the reaction was terminated by the addition of 1 mL of 1.25 m glycine for 5 min, followed by extracting the nuclear fraction using the hypotonic lysis buffer. Cross‐linked chromatin was transferred to AFA fiber tubes (Covaris) and sonicated in 0.2% SDS buffer. Sonicated chromatin was centrifuged for 5 min at 16 000 ***g*** and diluted to 0.1% SDS concentration. After preclearing with Dynabeads Protein G (Life Technologies, Carlsbad, CA, USA) for 1 h, chromatin–protein complexes were immunoprecipitated with 5 μg of antibodies overnight at 4 °C. The next day, Dynabeads Protein G was added for 2 h, and beads were washed in the buffer. Precipitated chromatin was then eluted from the beads in 300 μL elution buffer (1% SDS, 0.1 m NaHCO_3_) for 1 h at room temperature followed by de–cross‐linking at 65 °C overnight. After RNase A and proteinase K treatment, ChIP and input DNA were extracted by phenol‐chloroform extraction. Fragment sizes (200–300 bp) were evaluated by a High Sensitivity DNA Kit (Agilent Technologies, Santa Clara, CA, USA) on an Agilent 2100 Bioanalyzer. Specific enrichment was analyzed by qPCR and as percent input. For ChIP‐seq library preparation, ThruPLEX DNA‐seq Kit (Rubicon Genomics, Ann Arbor, MI, USA) was used with 2 ng of DNA input in 10 cycles of PCR amplification. The products were size‐fractionated and purified by a polyacrylamide gel. Fragment sizes and library concentrations were validated as above before sequencing on Illumina HiSeq 2500 (PE50) (illumina San Diego, CA, USA) at the MSKCC Integrated Genomics Operation core. ChIP‐seq raw data were mapped to the Human Genome 19 (NCBI build) by Bowtie using default parameters (This method is similar to that described in our previous paper [10]).

### RNA interference, DNA transfection, and lentiviral transduction

Sequences of short hairpin RNAs (shRNAs) used are listed in Table S1. siRNAs targeting genes of interest (ON‐TARGETplusTM siRNA) were purchased from Dharmacon (catalog numbers are listed in Table 1). The MYBL2 isoform plasmid was generously provided by S. Plymate (University of Washington, Seattle, WA), and the construct was subcloned into pHR′‐CMV‐GFP expression vector. Additional information can be found in Table S1.

**Table 1:** Characteristics of men with metastatic hormone‐sensitive prostate cancer in the CHAARTED trial with gene expression profiling of prediagnosis prostate biopsy by *MYBL2* level.^a^ ECOG, Eastern Cooperative Oncology Group; IQR, interquartile range; PSA, prostate‐specific antigen.

	MYBL2 mRNA expression level	
	High: Upper quartile	Low: All other
*n*	40	120
Age at randomization (years), median (IQR)	60 (53, 67)	64 (56, 69)
Race, *n* (%)		
White	37 (95)	106 (89)
Black	2 (5)	12 (10)
Other	0 (0)	1 (1)
Unknown	1	1
ECOG performance status, *n* (%)		
0	31 (78)	79 (66)
1	8 (20)	38 (32)
2	1 (3)	3 (3)
High‐volume disease, *n* (%)	31 (78)	94 (78)
Visceral disease, *n* (%)	24 (77)	75 (80)
Unknown	9	26
Gleason score ≥ 8, *n* (%)	34 (92)	90 (78)
Unknown/Missing	3	4
Baseline PSA (ng·mL^−1^), median (IQR)	67 (12, 167)	100 (26, 372)
Prior adjuvant hormone treatment, *n* (%)	0 (0)	4 (3)
Prior local treatment, *n* (%)		
None	40 (100)	101 (84)
Prostatectomy	0 (0)	12 (10)
Primary radiation	0 (0)	7 (6)

a Of 198 available samples, 190 passed pathology review, and 160 passed research assay metrics.

### Transfection experiments: qPCR and immunoblotting

Total RNA was isolated from cells using TRIzol (Invitrogen, Frederick, MD, USA) according to the manufacturer's protocol and quantified using a NanoDrop spectrophotometer. 1 μg of RNA was reverse‐transcribed using a High Capacity cDNA Reverse Transcription Kit (Applied Biosystems, Foster City, CA, USA). TaqMan gene expression assays (Life Technology, Carlsbad, CA, USA) were used for relative gene expression. Transcript levels were normalized to GAPDH. Proteins were extracted by radioimmunoprecipitation assay (RIPA) buffer with proteinase inhibitor mixture (Thermo Scientific, Carlsbad, CA, USA) and sonicated using a Bioruptor Standard for 5 min. RIPA buffer and protein concentration was determined by the Bradford method. Equal amounts of protein were loaded and resolved by SDS/PAGE, then transferred to polyvinylidene difluoride membranes for immunoblotting. All antibodies are listed in Table S1.

### Soft‐agar colony formation assay

A soft‐agar assay was performed in 6‐well cell culture plates in triplicates. 20 000 live cells (LNCaP sh‐control and overexpression of MYBL2, LAPC4 si‐control, and si‐MYBL2) were plated in 2 mL 0.3% Noble Agar mixture media onto 2 mL 0.6% Noble Agar media. Cells were incubated for 2 weeks, changing media every 3 days, and stained with 0.1% iodonitrotetrazolium chloride (INT) for 1 h. Colony numbers were counted and photographed by using Gel Count (Oxford Optronix, Oxford, UK).

### Western blot

Cells were washed with HBSS and lysed in radioimmunoprecipitation assay (RIPA) buffer unless otherwise noted (50 mm TRIS‐HCI pH 7.4, 150 mm NaCI, 1 mm EDTA, 1% Triton X‐100, 1% sodium deoxycholate, and 0.1% SDS) supplemented with protease and phosphatase inhibitors (ThermoFisher Scientific). Protein concentrations were measured using the Bradford protein assay. Western blot was performed using specific antibodies (Table S1). For BRCA2 western blot, we used Novex Tris‐Glycine Mini Gels, WedgeWell™ format (4–20%, ThermoFisher Scientific). The antibodies used are listed in Table S1. Western blot films in Fig. S1.

### Matrigel invasion assay

The matrigel invasion assays were performed in Matrigel invasion chambers (Fisher Scientific) in triplicates. 20 000 live cells (LNCaP sh‐control and overexpression of MYBL2) were plated on the top of chambers in serum‐free media. 10% FBS in the lower chamber was used as a chemo‐attractant. After 48 h incubation, cells in the bottom chamber were fixed in methanol and stained with crystal violet. Invaded cells were counted and photographed under phase‐contrast microscopy. In one chamber, colonies stained with crystal violet stain were visually counted.

### Apoptosis assay

Cells were cultured in 6‐well plates to 50% confluency, then transfected with siRNA. After 72 h, cells were collected in media with 1% FBS, mixed with Muse Annexin V and Dead Cell Reagent, and analyzed using a Muse Cell Analyzer (Millipore Sigma, Saint Louis, MO, USA).

### Cell cycle analysis

LNCaP‐wild‐type, lenti‐control plus si‐control, and overexpression of MYBL2 plus si‐KDM5D cells were cultured in a medium containing 10% CSS (Charcoal‐stripped serum). LAPC4‐wild‐type, lenti‐control plus si‐control, and overexpression of KDM5D plus si‐MYBL2 cells were cultured in 10% CSS medium for 72 h, and 1 million cells were harvested followed by washing with Hank's balanced salt solution (HBSS) and fixation with ice‐cold 70% ethanol overnight. Cells were then washed with HBSS, stained with 200 μL of PI/RNAse reagent (Millipore, Temecula, CA, USA) for 30 min, and the cell cycle distribution was analyzed using the Muse Cell Analyzer (Millipore).

### Gene expression profiling

Among men with mHSPC who participated in the CHAARTED trial, consent and IRB approval were obtained for genomic and gene expression studies. Formalin‐fixed paraffin‐embedded prostate biopsy tissues from before initiation of androgen deprivation therapy were profiled for whole‐transcriptome gene expression using the Affymetrix human exon 1.0 ST microarray (Decipher Biosciences, San Diego, CA, USA), as previously described [18, 19, 20]. In addition, gene expression data from previously published transcriptomic studies of prostate cancer in primary disease (Memorial Sloan Kettering Cancer Center series [21] and The Cancer Genome Atlas [TCGA] [22]), and in metastatic castration‐resistant disease (University of Michigan series [23], Fred Hutchinson Cancer series [24], StandUp2Cancer/Prostate Cancer Foundation Dream Team series [25]) were accessed via cBioPortal [26].

### Statistical analysis

Correlations of *KDM5D* and *MYBL2* levels were compared as continuous variables using Spearman correlation unless detailed otherwise in the results. Follow‐up for disease‐free survival including biochemical recurrence started at cancer diagnosis in cohorts of localized disease and randomization in CHAARTED. Overall survival and time to castration resistance were assessed using the Kaplan–Meier method and Cox proportional hazards regression. Patients who had tumors in the highest quartile of *MYBL2* expression were compared with outcomes of patients with lower *MYBL2*. In CHAARTED, multivariable models included ECOG performance status, disease volume (high *vs*. low, as defined in [27]), and *de novo* metastases *versus* relapse after local therapy. Each examination was performed in triplicate. For *in vitro* experiments, statistical significances of differences were evaluated by performing the two‐sided Student's *t*‐test. The values were presented as the mean ± standard deviation. A *P*‐value < 0.05 was considered to be statistically significant.

### Clinical specimens from the CHAARTED trial

Complete descriptions of clinical specimens from the CHAARTED trial of mHSPC have been published in [28]. In brief, Among the 190 available samples of formalin‐fixed, paraffin‐embedded (FFPE) biopsy and radical prostatectomy from the CHAARTED trial, 160 samples (84%) passed quality control for downstream analysis. Deidentified specimens were sent to Decipher Biosciences for central pathology review. The highest grade tumor focus was identified and underwent RNA extraction after macrodissection by a genitourinary pathologist. At least 0.5 mm^2^ of the tumor with at least 60% tumor cellularity was required for the assay. RNA was extracted using the RNeasy FFPE kit (Qiagen, Germantown, MD, USA), converted into cDNA and amplified using the Ovation FFPE kit (TECAN Genomics, Redwood City, CA, USA), and hybridized to the Human Exon 1.0 ST oligonucleotide microarray (Thermo Fisher), in a Clinical Laboratory Improvement Amendments‐certified laboratory facility (Decipher Biosciences). Quality control was performed using Affymetrix Power Tools, and normalization was performed using the single channel array normalization (SCAN) algorithm. The study methodologies conformed to the standards set by the Declaration of Helsinki. The experiments were undertaken with the understanding and written consent of each subject. The study methodologies were approved by the Dana‐Farber Harvard Cancer Centers.

## Results

### Correlation of KDM5D with MYBL2 in hormone‐sensitive prostate cancer

**Fig. 1:**
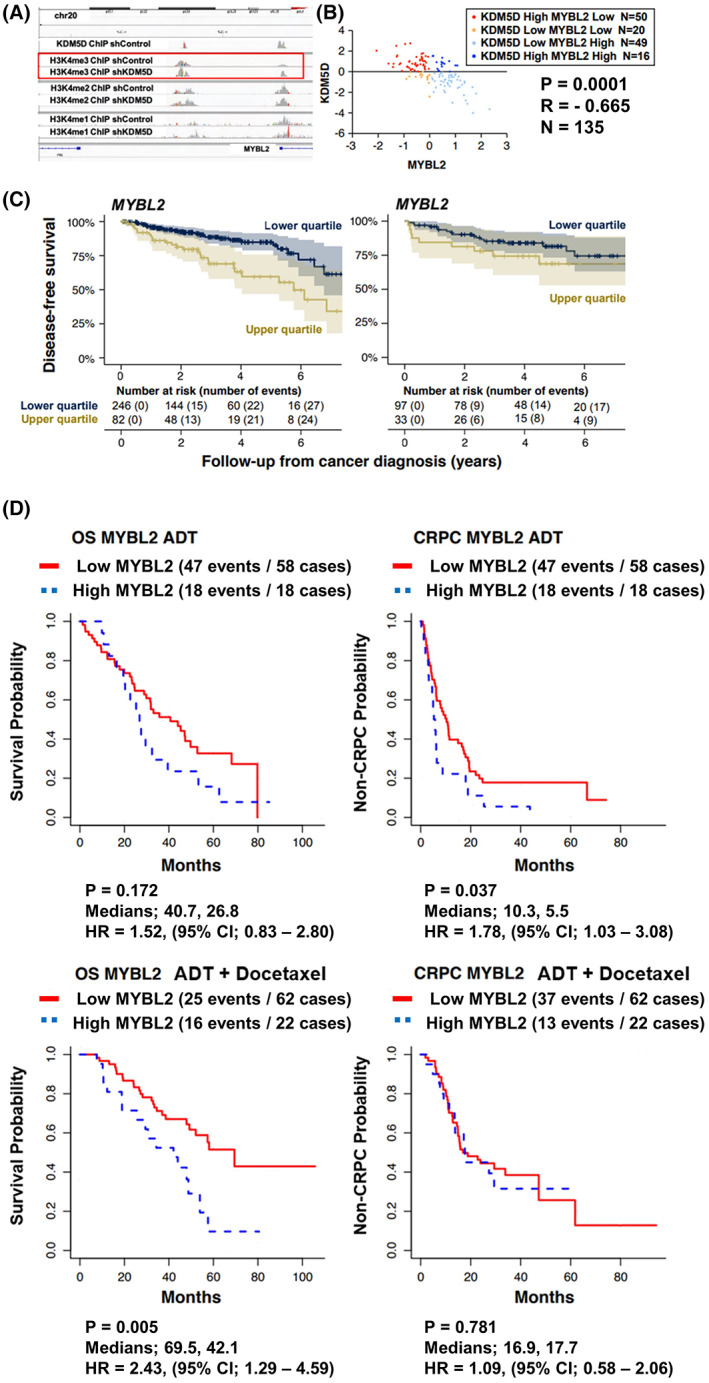
MYBL2 relationship with KDM5D in HSPC and association with clinical outcomes. ADT, androgen deprivation therapy; CRPC, castration‐resistant prostate cancer; OS, overall survival. (A) ChIP‐seq data of H3K4me1, 2, and 3 in LNCaP cells with and without silencing KDM5D. (B) Expression levels of KDM5D and MYBL2 in primary prostate cancer human samples (*n* = 135) (quoted from Taylor cohort C‐bioportal database). (C) Disease‐free survival based on the expression level of MYBL2 in localized prostate cancer (quoted from TCGA and Taylor cohorts); high: expression = above the median. (D) Time to CRPC and overall survival of patients from the CHAARTED trial based on levels of MYBL2 (MYBL2‐high, blue curves: upper quartile of gene expression; upper left; *n* = 76, *P* = 0.172, *P*‐value is from the log rank test/upper right; *n* = 76, *P* = 0.037, *P*‐value is from the log‐rank test/under left; *n* = 84, *P* = 0.005, *P*‐value is from the log‐rank test/under right; *n* = 84, *P* = 0.781, *P*‐value is from the log‐rank test).

Silencing *KDM5D* in the HSPC cell line LNCaP caused a marked increase in H3K4me3 peaks on ChIP‐seq but no major changes in H3K4me2 or H3K4me1 at the promotor region of *MYBL2* (Fig. 1A). Silencing KDM5D increased MYBL2 expression (Fig. S2A). This result was consistent with our prior RNAseq results of LNCaP and its androgen‐independent clone, LNCaP104R2, where an inverse correlation of expression between *KDM5D* and *MYBL2* was also observed [29].

We then quantified to what extent the negative expression correlation between *KDM5D* and *MYBL2* observed *in vitro* was reflected as an inverse association of *KDM5D* and *MYBL2* in tumor tissue from patients. Inverse correlations between *KDM5D* and *MYBL2* mRNA levels were observed in localized prostate cancer (*r* = −0.67, 95% CI –0.73 to −0.51; Fig. 1B) [21] and metastatic castration‐resistant prostate cancer (CRPC) (*r* = −0.24, 95% CI –0.42 to −0.03, in [23]; *r* = −0.26, 95% CI –0.40 to −0.12, in [24]). However, associations were null in cohorts of localized prostate cancer (*r* = 0.00, 95% CI –0.11 to 0.11, in [22]) and metastatic CRPC (*r* = −0.05, 95% CI –0.15 to 0.06, in [25]), and in the CHAARTED trial of mHSPC (Pearson *r* = −0.03, 95% CI −0.19 to 0.12).

### Elevated *MYBL2* is associated with poorer prognosis in hormone‐sensitive prostate cancer

High *MYBL2* expression was associated with a greater risk of relapse after local therapy (predominantly biochemical recurrence) in The Cancer Genome Atlas ([22], Fig. 1C), with a hazard ratio (HR) of 2.64 (95% confidence interval [CI], 1.55–4.49) for the upper quartile of mRNA expression compared with lower levels. This association was modestly attenuated when additionally adjusting for Gleason score (HR, 1.78; 95% CI, 1.00–3.16). In a smaller hospital‐based cohort of primary prostate cancer [21], an association of similar direction and magnitude was observed, although with less precision (Gleason‐adjusted HR, 1.87; 95% CI, 0.83–4.21).

We then studied the association of *MYBL2* and outcomes of men with mHSPC by profiling cancers from hormone‐naïve prostate gland specimens from 160 men in the CHAARTED trial (Table 1). The median follow‐up for this analytical cohort with available gene expression data was 4 years from randomization. A higher proportion of patients in the analytical population with successful transcriptome profiling had poor prognostic features than in the overall trial population, including *de novo* metastatic (88% *vs*. 73%) and high‐volume (78% *vs*. 65%) disease. As expected, the treatment effects of docetaxel were similar in the analytical population.

When analyzing the whole analytical population within CHAARTED regardless of treatment, OS was worse among men with *MYBL2*‐high tumors (31 deaths among men with tumors in the upper quartile of *MYBL2*) than among men with *MYBL2*‐low tumors (84 deaths for the lower three quartiles; unadjusted HR, 1.87; 95% CI, 1.21–2.89; Fig. S1A). However, the times to castration resistance for the 40 men with *MYBL2*‐high tumors (upper quartile; 31 events of CRPC), compared with the 120 men with MYBL2‐low tumors (lower three quartiles; 84 events) were not substantially higher (unadjusted HR, 1.35; 95% CI, 0.89–2.04; Fig. S3A).

Interestingly, among men treated with ADT only (Fig. 1D), high *MYBL2* expression tended to be more strongly associated with shorter time to CRPC (adjusted HR, 1.78; 95% CI, 1.03–3.08) than among men treated with ADT and docetaxel (adjusted HR, 1.09; 95% CI, 0.58–2.06; *P*_interaction_ by treatment arm, 0.058). By contrast, patients with MYBL2‐high tumors had poorer OS whether treated with ADT alone or ADT plus docetaxel. Specifically, there was no clear evidence of differences in associations of *MYBL2* with OS by treatment arm (*P*_interaction_, 0.42) with adjusted HR for high *MYBL2* of 1.64 (95% CI, 0.81–3.33) in the ADT‐only arm and of 2.71 (95% CI, 1.39–5.29) in the ADT plus docetaxel arm. High expression levels of MYBL2 were associated with a shorter time to recurrence and less effective treatment with ADT and docetaxel.

We also plotted the time to CRPC (left side Fig. S3B) and time from CRPC to prostate cancer or other death (right side) to provide a visual of the time in each disease state for each patient by *MYBL2* status and treatment arm. This facilitated an analysis of outcomes and potential treatment effects in the CRPC state with and without prior docetaxel given that OS is a composite of time in both states. Consistent with the modeling results above, patients with early docetaxel appear to have had a similar amount of time in the pre‐CRPC state regardless of *MYBL2* level. By contrast, patients with no early docetaxel and high *MYBL2* were more likely to have a short time in the pre‐CRPC state. Survival from the time of CRPC appeared similar regardless of MYBL2 levels and prior docetaxel.

Given our prior *in vitro* findings of KDMD5 and docetaxel [10], we also assessed the impact of KDM5D expression in CHAARTED samples. The only notable finding with the KDM5D analysis is that the group of men treated with ADT plus docetaxel and tumors with low *KDM5D* had a shorter time to CRPC than those with *KDM5D*‐high tumors when (adjusted HR: 2.73, 95%CI, 1.41–5.29), without a detectable association of *KDM5D* with OS (adjusted HR, 1.05; 95% CI, 0.50–2.20; Fig. S4).

### Elevated MYBL2 induces *in vitro* androgen resistance in prostate cancer cells

**Fig. 2:**
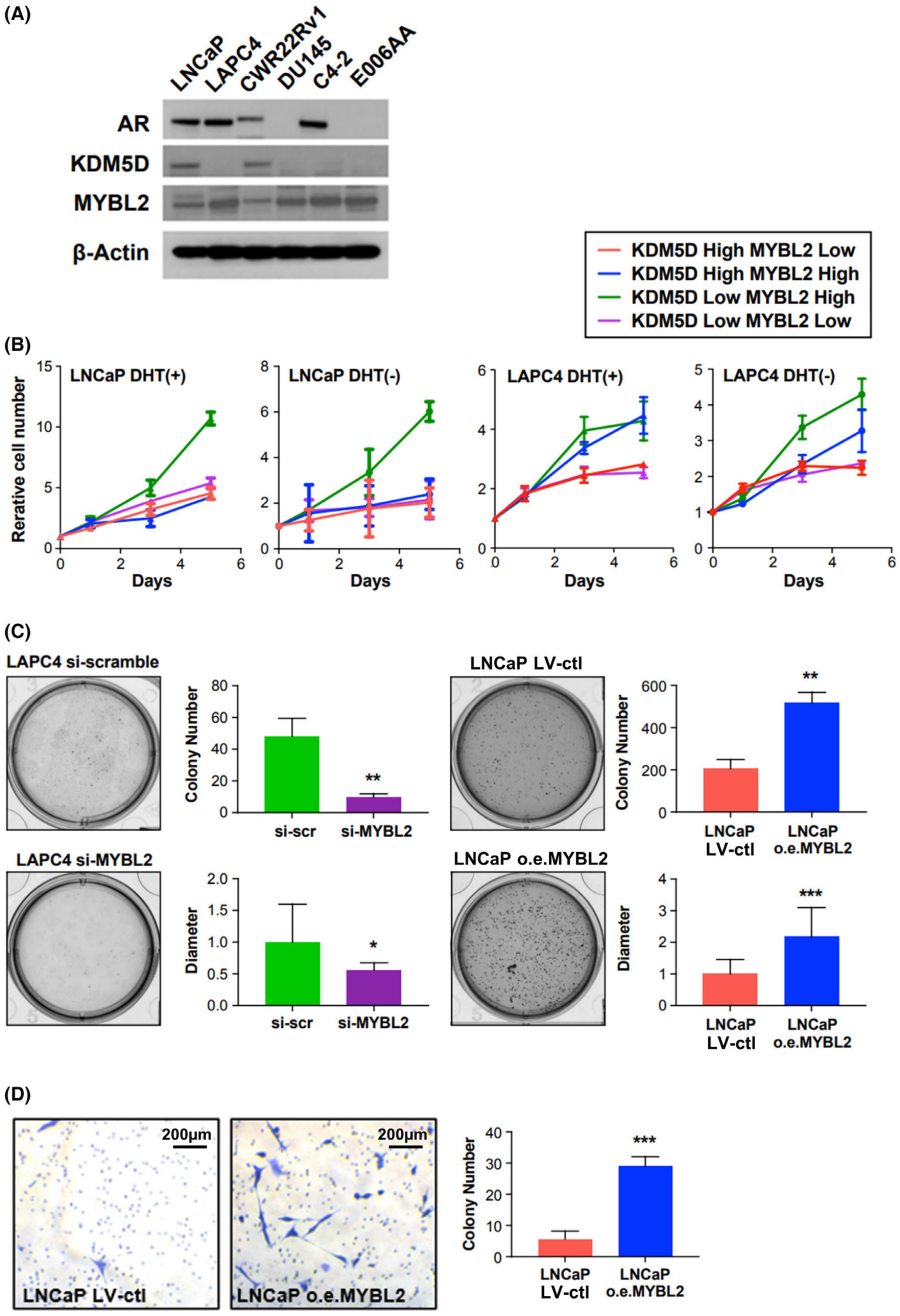
KDM5D‐low/MYBL2‐high promotes CRPC in this figure, all experiments were performed 3 times. AR, androgen receptor; CSS, charcoal‐stripped serum; ctl, control; DHT, dihydrotestosterone; LV, lentivirus; o.e., overexpression; scr, scramble. (A) Androgen receptor, KDM5D, and MYBL2 protein levels by western blot analysis of six prostate cancer cell lines. (B) Cell proliferation assay of indicated cell lines in CSS media with and without DHT. Error bars indicate standard deviation. LNCaP: KDM5D‐high/MYBL2‐low: si‐control/Lenti‐control; LNCaP: KDM5D‐high/MYBL2‐high: si‐*KDM5D* scramble/Lenti‐MYBL2; LNCaP: KDM5D‐low/MYBL2‐high: si KDM5D/Lenti‐MYBL2; LNCaP: KDM5D‐low/MYBL2‐low: si‐KDM5D/Lenti‐control; LAPC4: KDM5D‐high/MYBL2‐low: o.e.KDM5D + si‐MYBL2; LAPC4: KDM5D‐high/MYBL2‐high o.e.KDM5D + si‐control; LAPC4: KDM5D low/MYBL2‐high: Lenti‐control + si‐control; LAPC4: KDM5D low/MYBL2 low: Lenti‐control +si‐MYBL2. (C) Soft‐agar colony formation assay in LAPC4 si‐control, si‐MYBL2, LNCaP si‐control, and overexpression MYBL2. Representative images are shown after 14 days. The measured number of colonies and the measured diameter of the colony are represented in bar graphs. Error bars indicate standard deviation (*t*‐test; * indicates *P* < 0.05; **, *P* < 0.01; ***, *P* < 0.001). (D) Matrigel invasion assay in LNCaP lenti‐control, and overexpression MYBL2. Error bars indicate standard deviation (*t*‐test; *** indicates *P* < 0.001). The scale bar is 200 μm.

Assessments of the baseline protein expression levels of KDM5D and MYBL2 in a panel of prostate cancer cell lines (Fig. 2A) revealed the two cell lines with detectable KDM5D, LNCaP, and CWR22Rv1, had the lowest MYBL2 expression. To confirm the regulation of MYBL2 by KDM5D in the AR (+) androgen‐dependent cell lines, LNCaP and LAPC4, we silenced *KDM5D* in KDM5D‐high LNCaP cells and observed increases in *MYBL2* mRNA. Conversely, overexpressing *KDM5D* in LAPC4 cells decreased *MYBL2* RNA levels (Fig. S2A).

The influence of *KDM5D* and *MYBL2* expression on proliferation of the hormone‐sensitive cell lines LNCaP and LAPC4 was then assessed. The cell counts on Day 5 of wild‐type LNCaP‐KDM5D^Hi^/MYBL2^Lo^ (Fig. 2B; Fig. S2C) increased approximately fourfold in the presence of androgen‐replete medium (DHT(dihydrotestosterone)+) and less than 2‐fold in the absence of DHT an androgen‐deprived state (charcoal‐stripped medium without DHT) over 5 days. By contrast, overexpression of MYBL2 with silencing of *KDM5D* resulted in LNCaP: KDM5D^Lo^/MYBL2^Hi^ cell proliferation increasing by approximately 12‐fold in the presence of androgens and 6‐fold in the absence of DHT. However, when *KDM5D* was left intact with overexpression of *MYBL2*, the proliferation of LNCaP (KDM5D^Hi^/MYBL2^Hi^) cells increased approximately fourfold in the presence of DHT and about 2‐fold with androgen deprivation. The proliferation rate was also similar to wild‐type LNCaP when silencing *KDM5D* without silencing MYBL2 with a resultant modest increase but still lower MYBL2 levels than overexpressing MYBL2 in the LNCaP cells (KDM5D^Lo^/MYBL2^Lo^).

The findings from altering MYBL2 and KDM5D in the LAPC4 cell line were mostly consistent with those observed with LNCaP, despite their reversed constitutive expression levels (i.e., KDM5D^Lo^/MYBL2^Hi^ in wild‐type LAPC4). Namely, the LAPC4 KDM5D^Lo^/MYBL2^Hi^ cells lines had the most rapid proliferation in both DHT (+) and DHT (−) conditions whereas LAPC4 cells with KDM5D^Hi^/MYBL2^Lo^ and KDM5D^Lo^/MYBL2^Lo^ had the least proliferation in both DHT (+) and DHT (−) conditions. However, discordant with the LNCaP findings is that overexpressing *KDM5D* in LAPC4 cells with si‐control/no manipulation *MYBL2* did not slow proliferation in DHT (+) and partially slowed down the cellular proliferation in DHT (−) conditions, indicating more autonomy of MYBL2 from KDM5D in LAPC4.

We then further assessed the impact of MYBL2 on colony formation. Silencing *MYBL2* in LAPC4 (constitutively *MYBL2* ^Hi^) decreased colony formation both in number and size of colonies, whereas overexpressing *MYBL2* increased colony number and size in LNCAP cells (constitutively *MYBL2* ^Lo^; Fig. 2C). Overexpression of *MYBL2* in LNCaP increased invasiveness 6‐fold (Fig. 2D).

Having noted that the greater proliferation with and without androgen occurs in the low *KDM5D* and high *MYBL2* state, we further assessed the impact of silencing *MYBL2* alone in a dose‐dependent manner in LNCaP with intact KDM5D versus LAPC4 and E0006AA cells (both low *KDM5D* and high *MYBL2* expression). Whereas both low and high doses of siRNA had a minimal effect in LNCaP with minimal MYBL2, the higher siRNA doses caused an 80% and 90% inhibition of proliferation in LAPC4 and E006AA, respectively, and the lower siRNA dose had minimal effect in LAPC4 but an 80% inhibition in E006AA (Fig. S2C). We further confirmed the reliance of both LAPC4 and E0006AA cells with short hairpin RNA interference (Fig. S5A–D). Doxycycline induction of *MYBL2* shRNA on Day 2 also induced a subsequently marked inhibition of proliferation (Fig. S5E).

### Elevated MYBL2 decreases *in vitro* sensitivity to taxanes

**Fig. 3:**
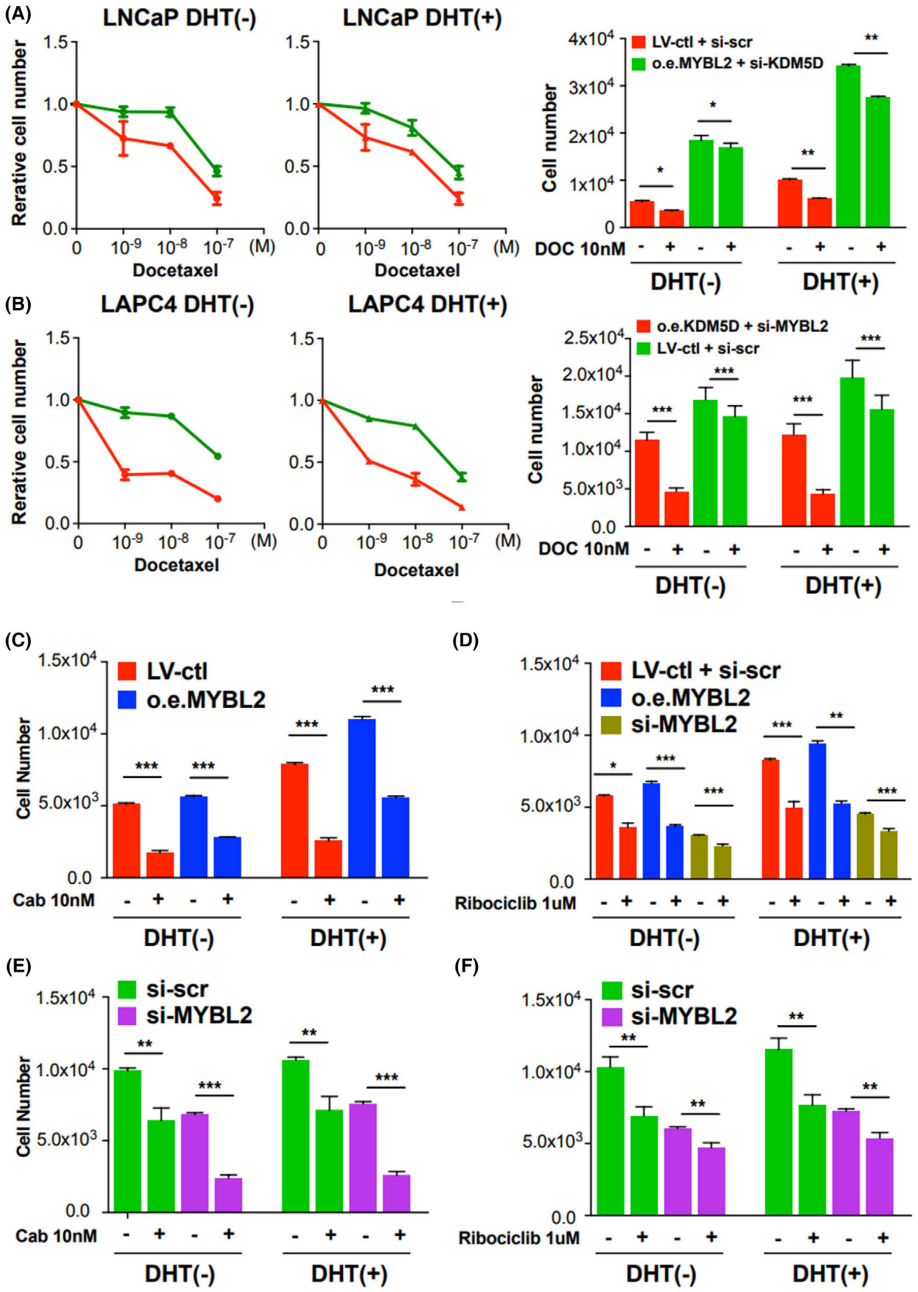
Impact of KDM5D and MYBL2 on sensitivity of hormone‐sensitive prostate cancer drugs targeting cell cycle apparatus. In this figure, all experiments were performed 3 times. Error bars indicate standard deviation (*t*‐test; * indicates *P* < 0.05; **P < 0.01; ***P < 0.001). ctl, control; DHT, dihydrotestosterone; DOC, docetaxel; LV, lentivirus; o.e., overexpression; scr, scramble. (a) LNCaP sensitivity to androgen deprivation and docetaxel *(LNCaP: Red = MYBL2* ^*Lo*^ */KDM5D* ^*Hi*^ *; green = MYBL2* ^*Hi*^ */KDM5D* ^*Lo*^ *)*. (B) LAPC4 sensitivity to androgen deprivation (left panel) and docetaxel (right panel). *(LAPC4: red = MYBL2* ^*Lo*^ */KDM5D* ^*Hi*^ *; green = MYBL2* ^*Hi*^ */KDM5D* ^*Lo*^ *)*. (C) LNCaP sensitivity to androgen deprivation and Cabazitaxel *(LNCaP: red = Lenti‐control = MYBL2* ^*Lo*^ */KDM5D* ^*Hi*^ *; blue = overexpression MYBL2 = MYBL2* ^*Hi*^ */KDM5D* ^*Hi*^ *)*. (D) LNCaP sensitivity to androgen deprivation and Ribociclib (CDK4/6 inhibitor). *(LNCaP: red = Lenti‐control = MYBL2* ^*Lo*^ */KDM5D* ^*Hi*^ *; blue = overexpression MYBL2 = MYBL2* ^*Hi*^ */KDM5D* ^*Hi*^ *; Brown = Lenti‐control plus si‐MYBL2 = MYBL2* ^*Lo*^ */KDM5D* ^*Hi*^ *)*. (E) LAPC4 sensitivity to androgen deprivation and Cabazitaxel *(LAPC4: Green = si‐control = MYBL2* ^*Hi*^ */KDM5D* ^*Lo*^ *; purple = si‐MYBL2 = MYBL2* ^*Lo*^ */KDM5D* ^*Lo*^ *)*. (F) LAPC4 sensitivity to androgen deprivation and Ribociclib (CDK4/6 inhibitor) *(LAPC4: Green = si‐control = MYBL2* ^*Hi*^ */KDM5D* ^*Lo*^ *; purple = si‐MYBL2 = MYBL2* ^*Lo*^ */KDM5D* ^*Lo*^ *)*

Given the observation that patients with tumors with lower *MYBL2* levels had the longest OS when treated with ADT plus docetaxel for mHSPC, we assessed the impact of MYBL2 on docetaxel *in vitro* sensitivity in the AR+ HSPC cell lines LNCaP and LAPC4 with *KDM5D* ^*Hi*^ */MYBL2* ^*Lo*^ and *KDM5D* ^*Lo*^ */MYBL2* ^*Hi*^ genotypes (Fig. 3A,B). Notably, both LNCaP and LAPC4 with the *KDM5D* ^*Hi*^ */MYBL2* ^*Lo*^ genotype (red curves) had a greater sensitivity to androgen deprivation, DHT (−) plus docetaxel than their counterparts with *KDM5D* ^*Lo*^ */MYBL2* ^*Hi*^ (green curves). The same observation was observed (middle panel Fig. 3A,B) with docetaxel with DHT (+).

When the absolute cell counts on Day 3 were assessed (right panel, Fig. 3A,B), LNCaP with *KDM5D* ^*Hi*^ */MYBL2* ^*Lo*^ (red bars) with and without DHT had about 1/3^rd^ the proliferation compared with LNCaP with *KDM5D* ^*Lo*^ */MYBL2* ^*Hi*^. The addition of docetaxel in DHT (−) and DHT (+) conditions resulted in the fewest number of cells on Day 3 in the LNCaP with *KDM5D* ^*Hi*^ */MYBL2* ^*Lo*^ whereas docetaxel had less of an effect in the more rapidly proliferating LNCaP‐*KDM5D* ^*Lo*^ */MYBL2* ^*Hi*^ cells (green bars) with and without DHT. Similarly, the addition of docetaxel to LAPC4 with *KDM5D* ^*Hi*^ */MYBL2* ^*Lo*^ showed caused 59% inhibition of cell proliferation whereas LAPC4 with *KDM5D* ^*Lo*^ */MYBL2* ^*Hi*^ showed only 13% inhibition of cell proliferation.

We then assessed the ‘intermediate’ genotypes. LNCaP with *MYBL2* overexpression with the native presence of KDM5D (i.e., *KDM5D* ^*Hi*^ */MYBL2* ^*Hi*^ genotype) were more sensitive to docetaxel without and with DHT (day 3 cell counts: ~ 0.6 and 1.5 × 10^4^; Fig. S6A) versus the LNCaP with KDM5D^Lo^/MYBL2^Hi^ (day 3 cell counts: ~ 1.5 and 2.5 × 10^4^; Fig. 3). Note these experiments were performed at the same time as those in Fig. 3. This is consistent with GEP CHAARTED data, where patients with tumors with higher *KDM5D* had a longer time to CRPC (more sensitive) with ADT plus docetaxel. When the expression of *MYBL2* in LAPC4 was decreased (and *KDM5D* not overexpressed), LAPC4 with *KDM5D* ^*Lo*^ */MYBL2* ^*Lo*^ were approximately as sensitive to docetaxel without and with DHT (day 3 cell counts: ~ 0.4 and 0.5 × 10^4^; Fig. S5B) as the LAPC4 with *KDM5D* ^*Hi*^ */MYBL2* ^*Lo*^ cells (day 3 cell counts: ~ 0.4 and 0.5 × 10^4^; Fig. 3), confirming the main driver of LAPC4 sensitivity being MYBL2 with no significant impact with KDM5D.

To corroborate the impact of MYBL2, we then assessed the efficacy of another taxane, we assessed the effect of cabazitaxel (Fig. 3C,E) on the cell lines with different *MYBL2* genotypes. Overexpressing *MYBL2* also decreased the sensitivity to cabazitaxel in DHT (−) and DHT (+) compared with their LNCaP control counterparts (Fig. 3C). Silencing *MYBL2* in LAPC4 also increased their sensitivity to cabazitaxel in DHT (−) and DHT (+) versus their control counterparts (Fig. 3E).

We then observed that higher *MYBL2* levels decreased the sensitivity of the LNCaP and LAPC4 cells in DHT (−) and DHT (+) to another class of agents interacting with cell cycle, a CDK4/6 inhibitor, ribociclib (Fig. 3D,F) but to a more modest degree than its impact on the taxanes. We also assessed the impact of MYBL2 on the efficacy of an ATR inhibitor based on our prior findings that loss of KDM5D expression leads to acceleration of the cell cycle and mitotic entry with increased DNA‐replication stress and that blocking ATR activity in KDM5D‐deficient cells induced DNA damage and apoptosis [29]. As seen in Fig. S6C, LNCaP *KDM5D* ^*Hi*^ */MYBL2* ^*Lo*^ cells proliferated more slowly and were more sensitive to the ATR inhibitor, VE‐822 at 100 nm than LNCaP *KDM5D* ^*Lo*^ */MYB2L* ^*Hi*^ cells in DHT (−) and DHT (+) conditions. In comparison, the LAPC4 (Fig. S6D), with *KDM5D* ^*Hi*^ */MBYL2* ^*Lo*^ and *KDM5D* ^*Lo*^ */MYBL2* ^*Hi*^ genotypes were both less sensitive to ATR inhibition this was not impacted by MYBL2 status.

### KDM5D and MYBL2 status impact of cell cycle and apoptosis

#### Impact on apoptosis

**Fig. 4:**
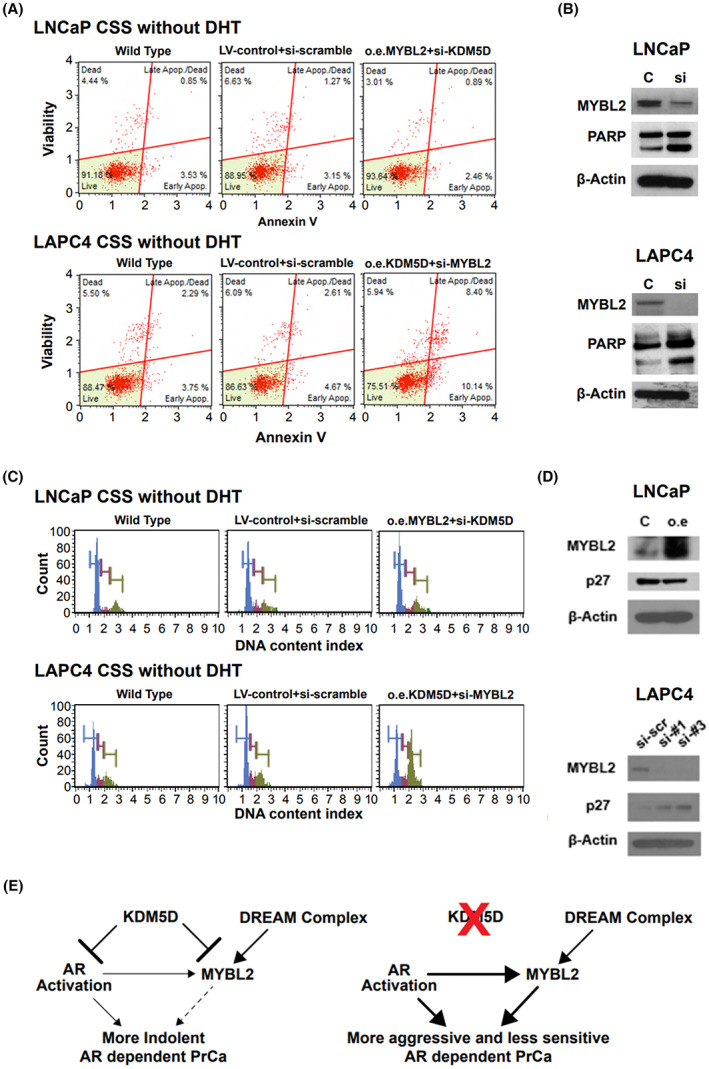
Impact of KDM5D and MYBL2 on apoptosis and cell cycling in hormone‐sensitive prostate cancer cells. In this figure from A to D, experiments were performed 3 times. AR, androgen receptor; CSS, charcoal‐stripped serum; DHT, dihydrotestosterone; LV, lentivirus; o.e., overexpression; PrCa, prostate cancer; scr, scramble. (A) Apoptosis activation was monitored in LNCaP and LAPC4 cells using muse apoptosis assay kit. (B) PARP protein levels were determined by western blot of LNCaP and LAPC4 control and knockdown MYBL2. (C) Cell cycle activation was monitored in LNCaP and LAPC4 cells using muse cell cycle. (D) Cell cycle‐related protein levels were determined by western blot of LNCaP and LAPC4. (E) Hypothetical schema showing the target genes of KDM5D/MYBL2 and its effects on prostate cancer networks

Having established that *KDM5D* ^*Hi*^ */MYBL2* ^*Lo*^ hormone‐sensitive AR‐positive cells have slower proliferation with DHT and greater sensitivity to androgen deprivation, we investigated the impact of KDM5D and MYBL2 expression on the distribution of dead, early and late apoptosis and live cells in the absence of DHTCSS (androgen deprivation). We noted (Fig. 4A) that the more rapidly proliferating LNCaP cells with silenced *KDM5D*/overexpressed *MYBL2* (i.e., *KDM5D* ^*Lo*^ */MYBL2* ^*Hi*^) had a slight increase in the percentage of live cells as well as fewer dead and apoptotic cells compared with wild‐type LNCaP and vector control (i.e., *KDM5D* ^*Hi*^ */MYBL2* ^*Lo*^). Consistent with this, we then noted that the more slowly proliferating LAPC4 cells with overexpressed KDM5D/silenced MYBL2 (i.e., *KDM5D* ^*Hi*^ */MYBL2* ^*Lo*^ *)* had a more notable increase in early and late apoptotic cells and much fewer live cells than the more rapidly proliferating *KDM5D* ^*Lo*^ */MYBL2* ^*Hi*^ LAPC4 cells (wild‐type and vector control). It therefore followed that silencing *MYBL2* (with the loss of its prosurvival/proliferation function) increased PARP cleavage in both the LNCaP and LAPC4 cells (Fig. 4B).

#### Impact on cell cycle

Given the increased proliferation in the *KDM5D* ^*Lo*^ */MYBL2* ^*Hi*^ hormone‐sensitive AR‐positive cells, we next determined their cell cycle distribution in CSS. We noted no major changes in the LNCaP *KDM5D* ^*Lo*^ */MYBL2* ^*Hi*^ cell line compared with the wild‐type and vector control. However, the slower proliferating LAPC4 cell line with overexpression of KDM5D/silenced MYBL2 had a notable decrease in the G0/G1 phase with a compensatory increase in G2/M (Fig. 4C). Having seen a major impact on cell cycle distribution in the LACP4 cells with loss of MYBL2, we interrogated key cell cycle proteins associated with silencing *MYBL2*. We observed overexpression of *MYBL2* in LNCaP resulted in a decrease in p27 with and an increase in p27 in the LAPC4 cells when MYBL2 was silenced consistent with the observed decrease of G0/G1 and increased G2/M in LAPC4 (Fig. 4D). Quantitative analysis of cell cycle profiles are in Table S2.

## Discussion

Having first identified that loss of the epigenetic factor KDM5D leads to more aggressive hormone‐sensitive prostate cancer *in vitro* and increased risk of relapse after treatment of localized disease, we endeavored to identify the pathways activated with the loss of KDM5D that lead to more rapid proliferation, and resistance to ADT and docetaxel [10, 29]. Herein, we detail that KDM5D loss leads to increased histone modifications in the promoter region of *MYBL2* with subsequent upregulation of MYBL2. Consistent with this, KMD5D and MYBL2 mRNA were inversely correlated in some cohorts. However, this correlation was not seen in other cohorts. This variability could be lost due to metastatic disease when losing portions of the Y chromosome with resultant in loss of KDM5D [10] and fewer specimens may have KDM5D to regulate MYBL2. Assay variability (RNAseq, Affymetrix array) and sample quality (FFPE, fresh frozen) and source of cancer (localized prostate cancer versus metastases) may also contribute to the lack of consistent findings. Further work with uniform assays and samples is needed to clarify this finding [23, 24, 30, 31, 32]. Furthermore, the lack of consistent correlation between KDM5D and MYBL2 supports the hypothesis, and the literature detailing MYBL2 is also controlled by mechanisms (Fig. 4E) other than KDM5D, such as the DREAM multiprotein complex (dimerization partners, RB‐like proteins, E2Fs, MuvB core) [33, 34, 35, 36]. MYBL2 is a transcription factor of the MYB transcription factor family that regulates cell cycle progression, cell survival, and cell differentiation [37]. During the S phase, the MuvB core recruits MYBL2 and FOXM1 to increase expression of late cell cycle gene expression [33, 35].

The clinical relevance of elevation of *MYBL2* in prostate cancer was seen by the fact that patients with higher *MYBL2* had a higher rate of relapse after treatment of localized disease. Moreover, the patients with mHSPC treated with ADT alone and who had elevated *MYBL2* had a significantly shorter time to CRPC and this was associated with a nonsignificant 52% increase rate of death.

Interestingly, patients with elevated *MYBL2* had a shorter time to CRPC when treated with ADT alone, but not when treated with ADT plus docetaxel. However, the data for potential interactions between *MYBL2* expression and docetaxel treatment were inconclusive for overall survival. If observations on castration resistance were confirmed in future studies and corroborated by overall survival data, then patients with MYBL2‐high tumors might be preferentially considered for early docetaxel treatment. At this time, the data indicate that low *MYBL2* is a favorable prognostic marker, but it is not ripe for use as a predictive biomarker for treatment selection.

Consistent with our prior *in vitro* work that hormone‐sensitive cells with low KDM5D were less sensitive to docetaxel [10], men treated with ADT plus docetaxel who had tumors and low *KDM5D* had a shorter time to CRPC than those with *KDM5D*‐high tumors (adjusted HR: 2.73, 95%CI, 1.41–5.29). We did not detect an association with overall survival. The finding that elevation of *MYBL2* had a more consistent association with poorer mHSPC outcomes than lower *KDM5D* is consistent with *MYBL2* being a transcription factor controlled by other factors that promote many of the hallmarks of cancer [17]. The sample size did not permit analyzing outcomes by *MYBL2* and *KDM5D* status jointly.

The *in vitro* cell line work was largely consistent with the observational data among patients. Specifically, AR+ HSPC cell lines, LNCaP and LACP4 and their derivatives with KMD5D^Hi^/MYBL2^Lo^ genotypes, and compared with their KMD5D^Lo^/MYBL2^Hi^ counterparts, that had the most rapid proliferation, were the least sensitive to androgen deprivation and had the least sensitivity to docetaxel with and without DHT. The intermediate genotypes of KMD5D^Lo^/MYBL2^Lo^ and KMD5D^Hi^/MYBL2^Hi^ showed that the presence of KDM5D modulated the impact of MYBL2 in LNCaP but not LAPC4. One explanation is that there is a little constitutive expression of KDM5D in LAPC4, and it could suggest that the MYBL2 effects may be independent from KDM5D to some degree [34] (see schema in Fig. 4E). As such, MYBL2 is a notable but not the sole driver of the effects of KDM5D, and KDM5D influences MYBL2 in some but not all HSPC. Such heterogeneity may also contribute to the observed heterogeneity in associations between *KDM5D* and *MYBL2* in clinical specimens.

Finally, we characterized the type of cell death and cell cycle distribution upon silencing of *MYBL2*. In brief, in both androgen‐dependent cell lines there were more ‘live’ cells by FACS in the more aggressive variants with KDM5D^Lo^/MYBL2^Hi^ compared with their vector controls. In both cell lines, silencing MYBL2 increased PARP levels, consistent with the loss of a transcription factor promoting cell survival. The cell cycle distribution analysis revealed no appreciable difference between LNCaP cells with KDM5D^Hi^/MYBL2^Lo^ versus KDM5D^Lo^/MYBL2^Hi^ whereas overexpressing KDM5D with downregulation of MYBL2 in LAPC4 substantially decreased G0/G1 and increased G2/M. The cell cycle proteins associated with silencing MYBL2 in LAPC4 showed increased Cyclin A and p72, which are proteins associated with transitioning from G0/G1 to G2/M [38]. Notably, the greater increase of G2/M with KDM5D^Hi^/MYBL2^Lo^ genotype in LAPC4 was associated with its greater sensitivity (Fig. 3A and B) to docetaxel (IC_50_ ~ 1 nm) than was seen with LNCaP (IC_50_ ~ 50 nm). By contrast, LNCaP KDM5D^Hi^/MYBL2^Lo^ had greater sensitivity to the ATR inhibitor, which was partially abrogated by increasing MYBL2 levels but had no effect on LAPC4.

The limitation of our work is the paucity of mechanistic data and the limited experimental model systems. Findings in this study are supported by recent work by Li et al. [16] who showed MYBL2 to be upregulated in CRPC tissue and cell lines; in their study, overexpression of MYBL2 resulted in *in vitro* and *in vivo* castration‐resistant growth and metastases by activating YAP1. The association between high *MYBL2* levels and worse clinical outcomes across several cohorts that we examined and our corroborating *in vitro* findings suggest that further mechanistic studies exploring the cellular machinery driven by MYBL2 activation might lead to actionable insights on the progression of mHSPC.

Taken together, high MYBL2 is consistently associated with poorer patient prognosis in localized prostate cancer and mHSPC and leads to more aggressive cell lines and resistance to androgen deprivation and anti‐cancer therapies impacting cellular replication. Increased MYBL2 is a poor prognostic marker for patients with mHSPC with ADT treatment alone and ADT plus alone or with docetaxel, with the potential as a predictive biomarker for treatment selection if confirmed by future studies. The development of an MYBL2 inhibitor for clinical use may improve the efficacy of ADT and ADT with docetaxel for the treatment of mHSPC.

## Conclusions

The transcription factor *MYBL2* impacts both *in vitro* hormone‐sensitive prostate cancer sensitivity to androgen deprivation and taxanes and lower levels are associated with better clinical outcomes in men with hormone‐sensitive prostate cancer.

## Conflict of interest

Philip Kantoff: As of August 15th, 2021, PWK reports the following disclosures for the last 24‐month period: he has an investment interest in Convergent Therapeutics Inc, Cogent Biosciences, Context Therapeutics LLC, DRGT, Mirati, Placon, PrognomIQ, SnyDevRx, and XLink, he is a company board member for Context Therapeutics LLC and Convergent Therapeutics, he is a company founder for XLink and Convergent Therapeutics and is/was a consultant/scientific advisory board member for Anji, Candel, DRGT, Immunis, AI (previously OncoCellMDX), Janssen, Progenity, PrognomIQ, Seer Biosciences, SynDevRX, Tarveda Therapeutics, and Veru, and serves on data safety monitoring boards for Genentech/Roche and Merck. He reports spousal association with Bayer. Christopher Sweeney, MBBS: Consulting or Advisory Role: Sanofi, Janssen, Astellas Pharma, Bayer, Genentech, Pfizer, Lilly, Research Funding: Janssen Biotech (Inst), Astellas Pharma (Inst), Sanofi (Inst), Bayer (Inst), Sotio (Inst), Dendreon (Inst); Patents, Royalties, Other Intellectual Property: Parthenolide (Indiana University): dimethylaminoparthenolide (Leuchemix); Exelixis: Abiraterone plus cabozantinib combination. Stock or other ownership: Leuchemix;

## Author contributions

YY carried out the experiment with support from YM, GC, SHR, and RH. KHS, XVW, and YHC developed the theoretical formalism, performed the analytic calculations, and performed the numerical simulations. FB, SN, DF, GL, and MAC analyzed the data. ED and GSML were involved in planning this project. YY wrote the manuscript with support from CJS. Both HA and PWK conceived the study and were in charge of overall direction and planning. CJS supervised the project. All authors provided critical feedback and helped shape the research, analysis, and manuscript. All authors discussed the results and contributed to the final manuscript.

### Peer review

The peer review history for this article is available at https://publons.com/publon/10.1002/1878‐0261.13314.

## Supporting information


**Fig. S1.** Western blot sheet.
**Fig. S2.** Cell lines phenotype silencing MYBL2.
**Fig. S3.** Clinical outcomes in CHAARTED.
**Fig. S4.** Impact of KDM5D and MYBL2 on time to CRPC and OS in CHAARTED.
**Fig. S5.** Doxycycline‐induced sh‐MYBL2.
**Fig. S6.** Impact of KDM5D and MYBL2 on sensitivity to drugs in HSPC.
**Table S1.** Materials.
**Table S2.** Quantitative analysis of cell cycle profiles.

## Data Availability

Data availability statement gene expression profile data from the CHAARTED trial are available at: https://www.ncbi.nlm.nih.gov/geo/query/acc.cgi?acc=GSE201805
