# Supplementary material for: Increased MYBL2 expression in aggressive hormone‐sensitive prostate cancer
Source: Mol Oncol. 2022 Oct 2;16(22):3994–4010. doi: 10.1002/1878-0261.13314 (PMC9718114; doi:10.1002/1878-0261.13314)
Supplement: Supplementary file 1 — Fig. S1. Western blot sheet. Fig. S2. Cell lines phenotype silencing MYBL2. Fig. S3. Clinical outcomes in CHAARTED. Fig. S4. Impact of KDM5D and MYBL2 on time to CRPC and OS in CHAARTED. Fig. S5. Doxycycline‐induced sh‐MYBL2. Fig. S6. Impact of KDM5D and MYBL2 on sensitivity to drugs in HSPC. Table S1. Materials. Table S2. Quantitative analysis of cell cycle profiles. [file MOL2-16-3994-s001.pdf]

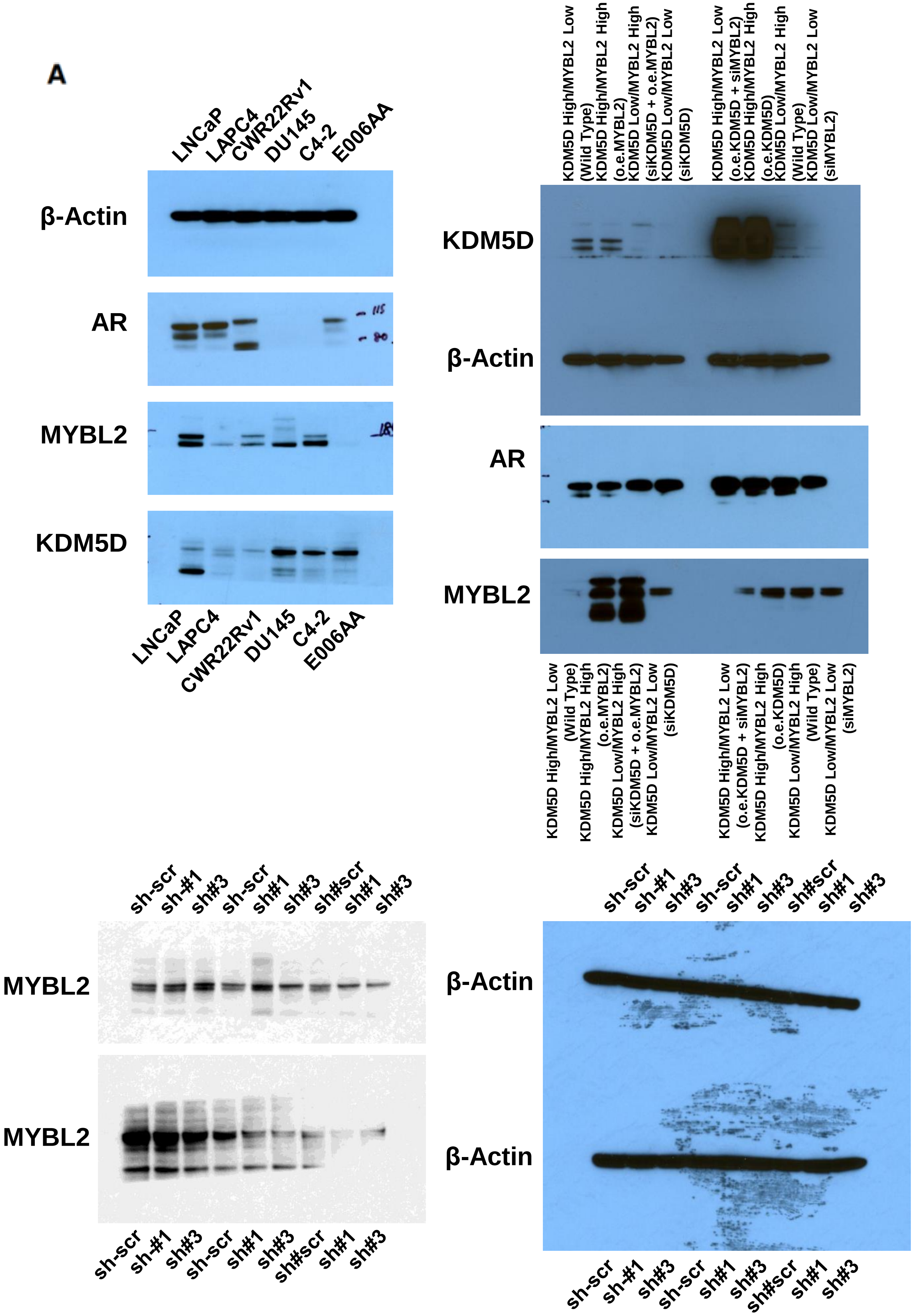

Supplementary Figure 1

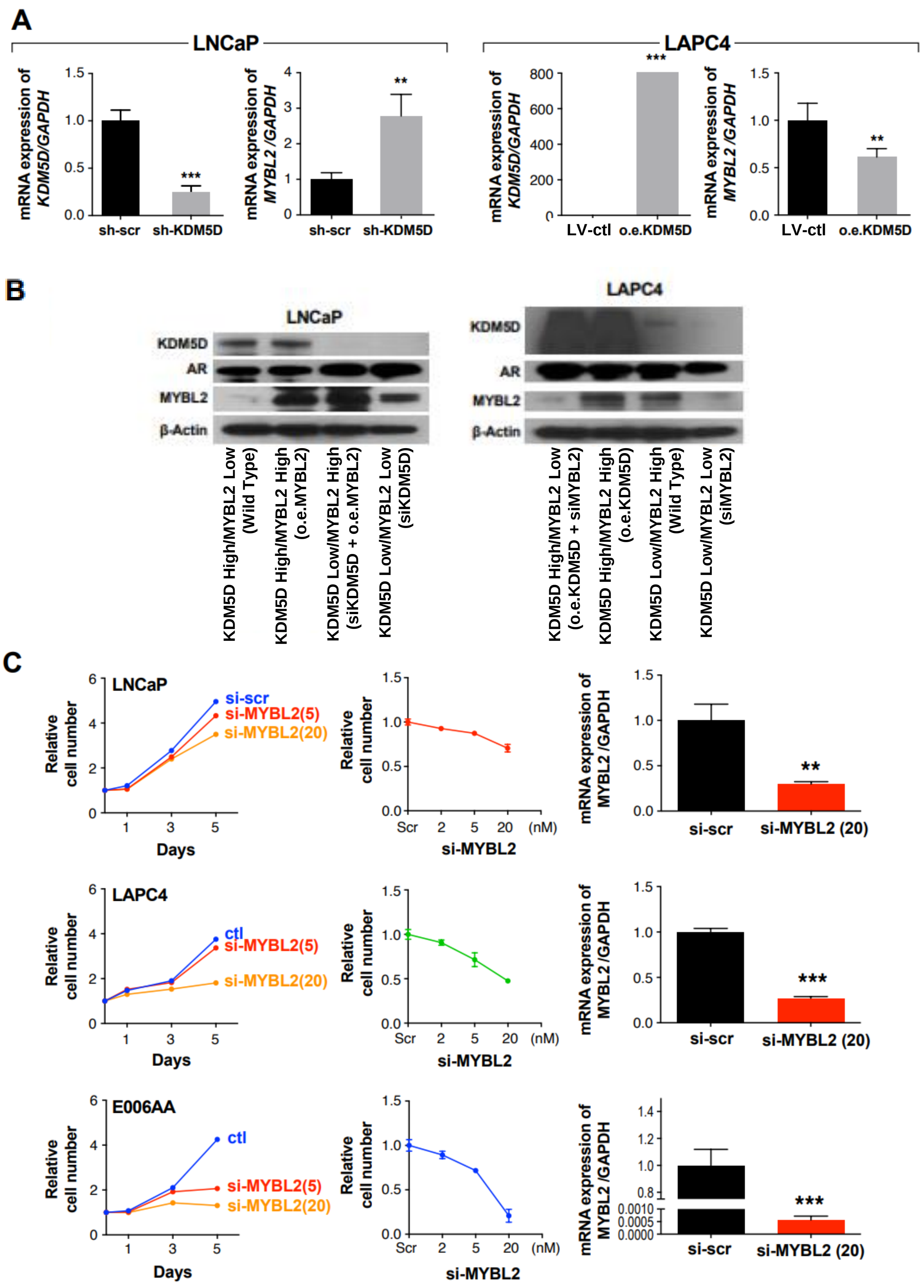

Supplementary Figure 2

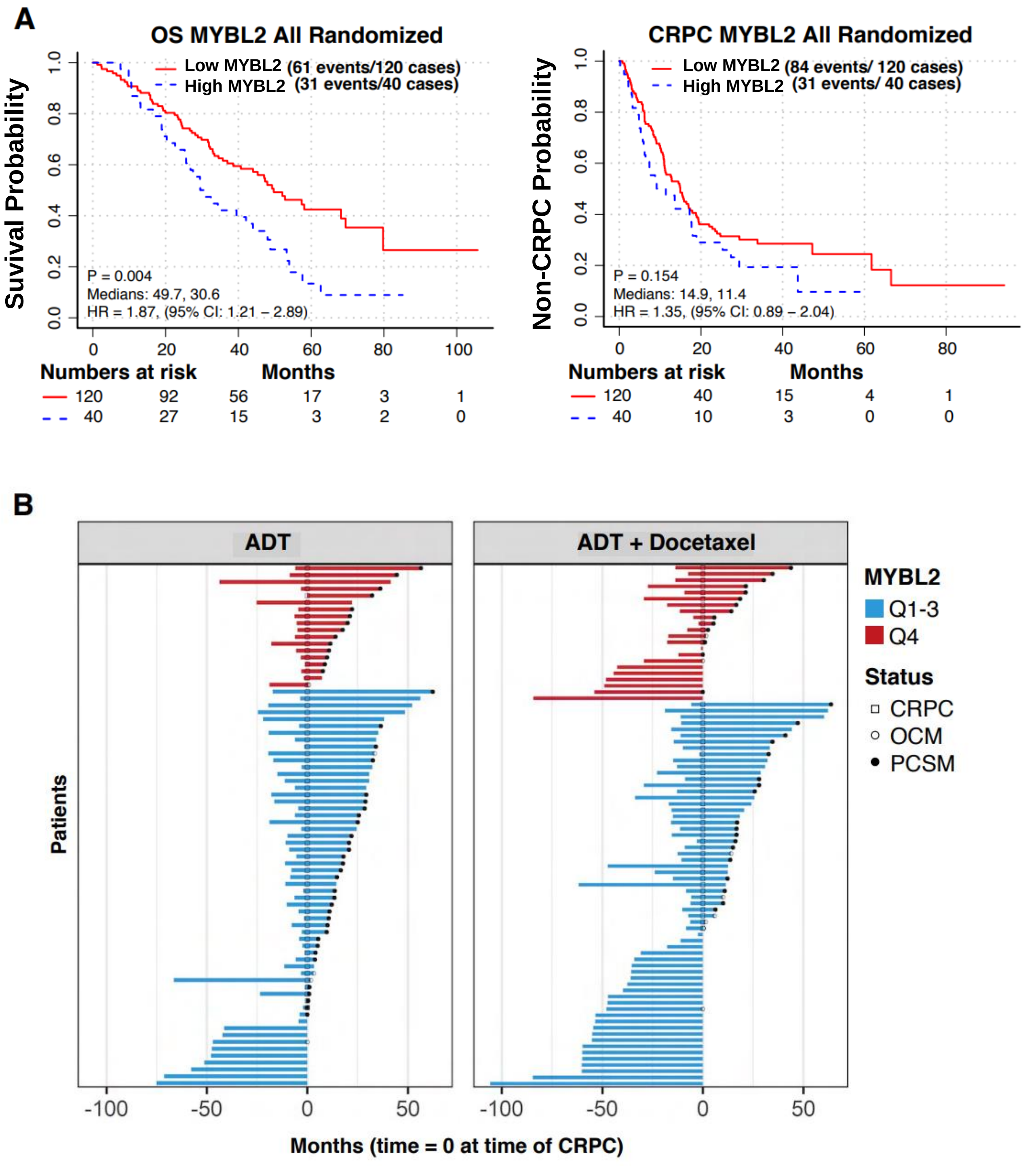

Supplementary Figure 3

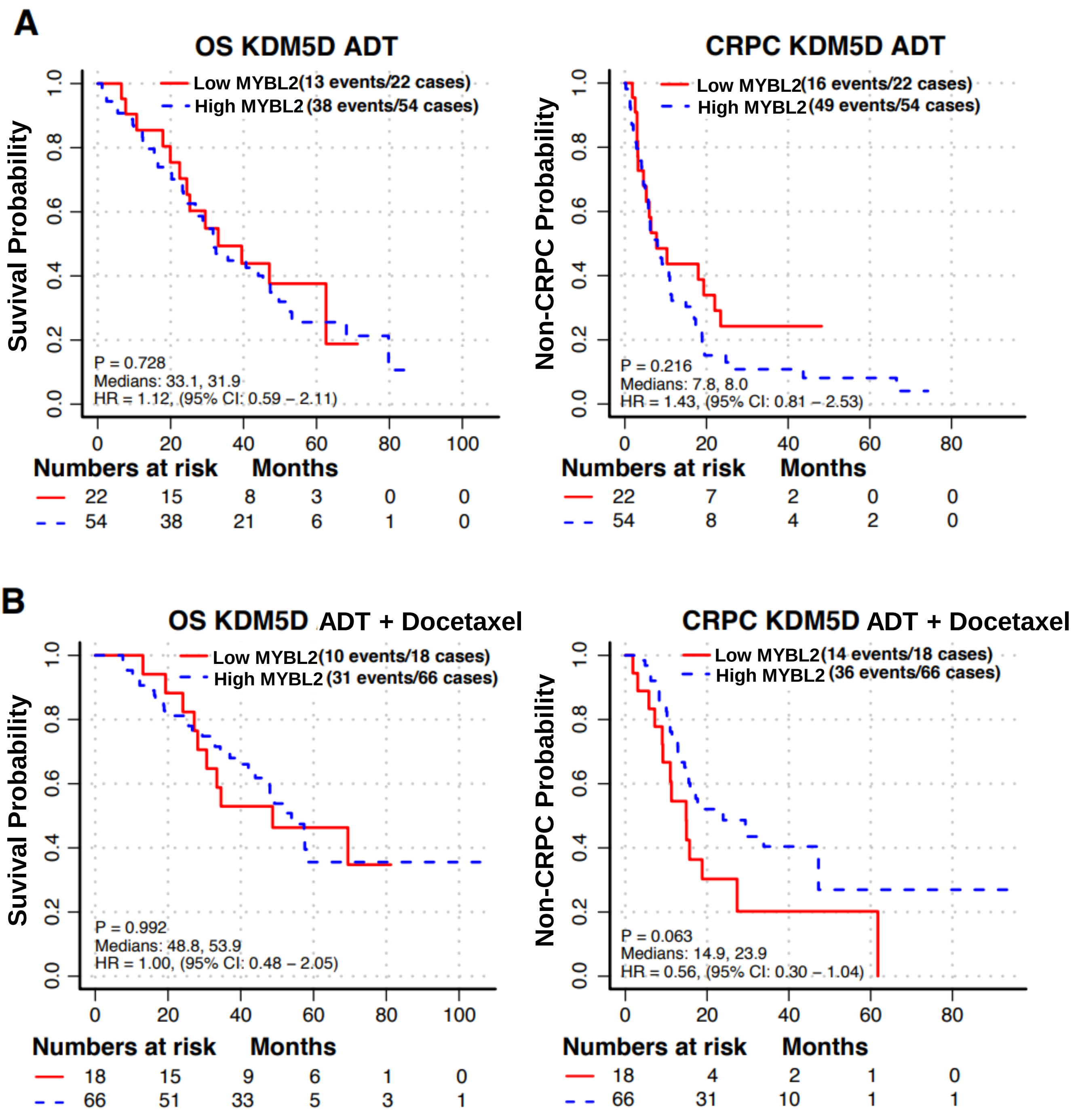

Supplementary Figure 4

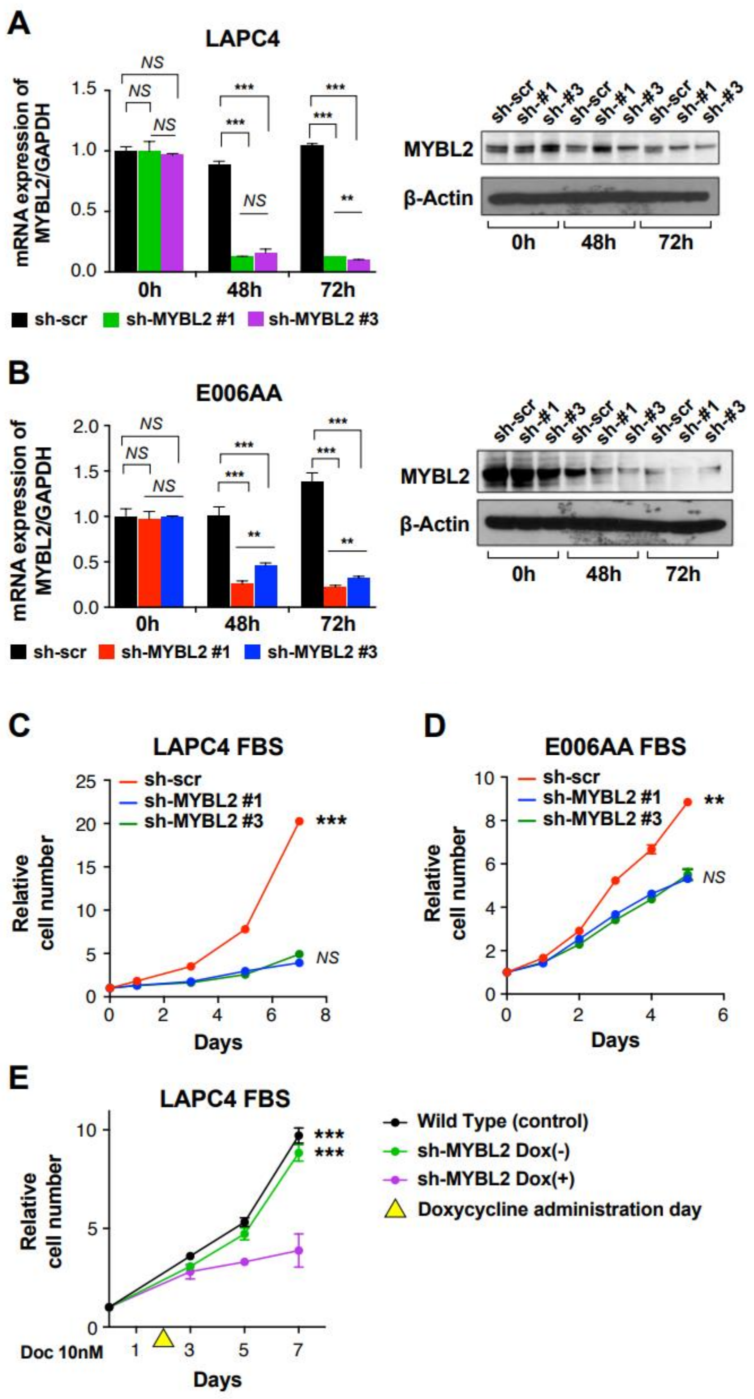

Supplementary Figure 5

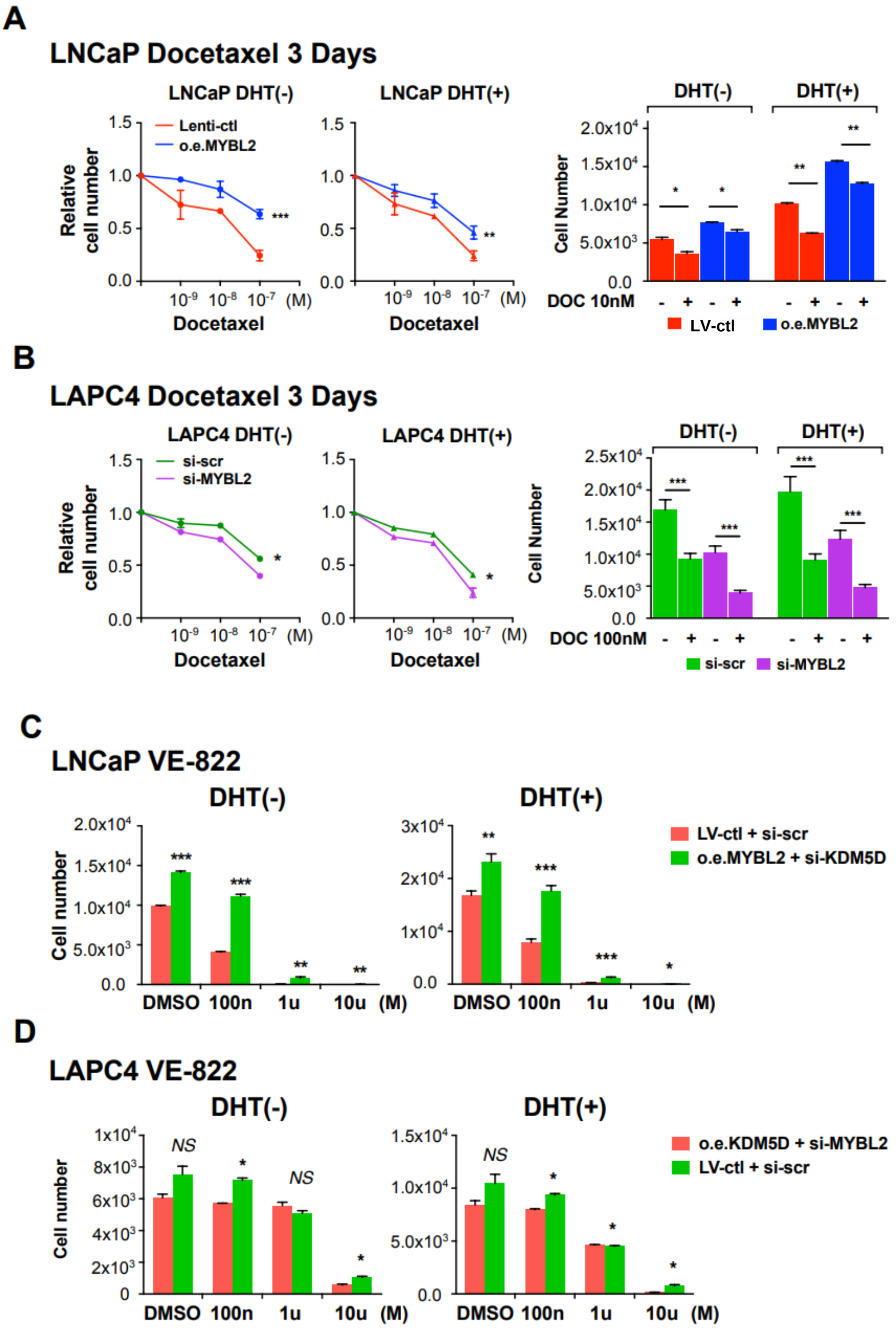

Supplementary Figure 6

Supporting Information section

Supplementary Figure Legends

Supplementary Figure 1: Western blot sheet
(A) Western blot sheet
Upper left; The protein expression levels of KDM5D, β-Actin, AR, and MYBL2 in LNCaP, LAPC4, CWR22Rv1, DU145, C4-2, and E006AA cell lines.
Upper right; The protein expression levels of KDM5D, β-Actin, AR, and MYBL2 in LNCaP and LAPC4 (each cells shows High/ Low KDM5D and High/ Low MYBL2) cell lines.
Under left; The protein expression levels of MYBL2 in sh-MYBL2 in LNCaP and E006AA.
Under right; The protein expression levels of β-Actin in sh-MYBL2 in LNCaP and E006AA.

Supplementary Figure 2: Cell lines phenotype silencing MYBL2
(A) mRNA expression level of KDM5D and MYBL2 in LNCaP (sh-control and sh-KDM5D) and LAPC4 (sh-control and over expression KDM5D) with FBS media. Error bars indicate standard deviation. (t-test; ** indicates $p < 0.01$; ***, $p < 0.001$).
(B) Western blot analysis of KDM5D, AR and MYBL2 levels corresponding to the RNA manipulations for Fig 2B after overexpressing or silencing KDM5D and/or MYBL2 as indicated
(C) Cell proliferation silencing MYBL2 (si-control, si-MYBL2 (5nM, 20nM)) with FBS media in three kind of cell lines. Cell proliferation after 3 days induced si-MYBL2 dose dependent (si-control(20nM), si-MYBL2 (2nM, 5nM, 20nM)) in three kind of cell lines. mRNA expression levels of MYBL2 by silencing MYBL2. Error bars indicate standard deviation. (t-test; ** indicates $p < 0.01$; ***, $p < 0.001$).

Supplementary Figure 3: Clinical outcomes in CHAARTED
(A) Clinical outcomes time to CRPC and OS related with MYBL2 expression level in all treatment from CHAARTED. (High = upper quartile)
(B) Time to CRPC and time from CRPC to death by treatment arm and MYBL2 status.
Abbreviations: CRPC, castration resistant prostate cancer; OCM, other cause mortality; PCSM, prostate cancer specific mortality; Q1, lowest quartile; Q2-3, middle quartiles; Q4, highest quartile.

Supplementary Figure 4: Impact of KDM5D and MYBL2 on time to CRPC and OS in CHAARTED
(A) Clinical outcomes time to CRPC from CHAARTED by KDM5D levels (Low = lower quartile).
(B) Clinical outcomes time to OS from CHAARTED by KDM5D levels (Low = lower quartile).

Supplementary Figure 5: Doxycycline induced sh-MYBL2
Error bars indicate standard deviation. (t-test; ** indicates $p < 0.01$; ***, $p < 0.001$).
(A) The time course of mRNA expression levels of MYBL2 in LAPC4 sh-control, sh-MYBL2#1, and sh-MYBL2#3 after administrated doxycycline 0 hour, 48 hours, and 72 hours.
(B) The time course of mRNA expression levels of MYBL2 in E006AA sh-control, sh-MYBL2#1, and sh-MYBL2#3 after administrated doxycycline 0 hour, 48 hours, and 72 hours.
(C) Cell proliferation assay of indicated cell lines in FBS media. LAPC4 sh-control vs sh-MYBL2#1 and #3.
(D) Cell proliferation assay of indicated cell lines in FBS media. E006AA sh-control vs sh-MYBL2#1 and #3.

(E) Cell proliferation assay of indicated cell lines with doxycycline inducible MYBL2 in FBS media. LAPC4 sh-control vs sh-MYBL2#1 and #3 with doxycycline treatment.

Supplementary Figure 6: Impact of KDM5D and MYBL2 on Sensitivity to drugs in HSPC
Error bars indicate standard deviation. (t-test; * indicates $p < 0.05$; **, $p < 0.01$; ***, $p < 0.001$).
(A) Docetaxel sensitivity Lenti-control *(=KDM5D^{Hi} / MYBL2^{Lo})* vs overexpression MYBL2 *(=KDM5D^{Hi} / MYBL2^{Hi})* in LNCaP with CSS plus DHT without DHT.
(B) Docetaxel sensitivity si-control *(=KDM5D^{Lo} / MYBL2^{Hi})* vs si-MYBL2 *(=KDM5D ^{Lo} / MYBL2^{Lo})* in LAPC4 with CSS plus DHT without DHT.
(C) Drug sensitivity of VE-822 (ATR-inhibitor) Lenti-control plus si-control *(=KDM5D^{Hi} / MYBL2^{Lo})* vs overexpression MYBL2 plus si-KDM5D (=*KDM5D^{Lo} / MYBL2^{Hi})* in LNCaP with CSS plus DHT without DHT.
(D) Drug sensitivity of VE-822 (ATR-inhibitor) Lenti-control plus si-control *(=KDM5D^{Lo}/MYBL2^{Hi})* vs overexpression KDM5D plus si-MYBL2 *(=KDM5D^{Hi} /MYBL2^{Lo})* in LAPC4 with CSS plus DHT without DHT.

Supplementary Table 1: Materials

Protein	Antibody	Vendor	Catalog Number
Androgen Receptor	Rabbit	Cell Signaling Technology	#5153
KDM5D / Jarid1D	Rabbit	Abcam	ab227728
B-Myb	Rabbit	Novus Biologicals	NBP2-33930
β-Actin	Mouse	Santa Cruz Biotechnology	sc-47778
PARP	Rabbit	Cell Signaling Technology	#9542
CyclinA	Mouse	Santa Cruz Biotechnology	sc-271682
p27 Kip1	Rabbit	Cell Signaling Technology	#2552

RNA(Primer)	Target		Catalog Number
MYBL2	Human	Hs00942540_m1	4331182
MYBL2	Human	Hs00942547_m1	4351372
MYBL2	Human	Hs00942552_m1	4351372
MYBL2	Human	Hs00942541_m1	4351372
MYBL2	Human	Hs00942551_m1	4351372
MYBL2	Human	Hs00942549_m1	4351372
KDM5D	Human	Hs00190491_m1	4331182
KDM5D	Human	Hs05049162_s1	4351372
KDM5D	Human	Hs01104401_g1	4351372
KDM5D	Human	Hs01104405_gH	4351372
KDM5D	Human	Hs01104389_g1	4351372
KDM5D	Human	Hs01104397_gH	4331182
GAPDH	Human	Hs99999905_m1	4331182

siRNA	Target	Vendor	Catalog Number
si-MYBL2	Human	Dharmacon	L-010444-00-0005
si-KDM5D	Human	Dharmacon	L-010820-00-0005

Overexpression			
B-MYB		Origene	RC203540L3V
KDM5D		Dharmacon	MHS6278-213246113

shRNAs

sh-scramble (control)

Forward:

CCGGTTTCTCCGAACGTGTCACGTCTCGAGACGTGACACGTTCGGAGAATTTTTG

Reverse:

AATTCAAAAATTCTCCGAACGTGTCACGTCTCGAGACGTGACACGTTCGGAGAAA

sh-KDM5D

Forward:

CCGGTGTAACACACACCCGTTTGACACTCGAGTGTCAAACGGGTGTGTGTTACTTTTTG

Reverse:

AATTCAAAAAGTAACACACACCCGTTTGACACTCGAGTGTCAAACGGGTGTGTGTTACA

sh-MYBL2 #1

Forward:

CCGGTGCTTCTTGAGCGAGTCCAAAGCTCGAGCTTTGGACTCGCTCAAGAAGCTTTTTG

Reverse:

AATTCAAAAAGCTTCTTGAGCGAGTCCAAAGCTCGAGCTTTGGACTCGCTCAAGAAGCA

sh-MYBL2 #3

Forward:

CCGGTGACACAGGAGGCTTCTTGAGCCTCGAGGCTCAAGAAGCCTCCTGTGTCTTTTTG

Reverse:

AATTCAAAAAGACACAGGAGGCTTCTTGAGCCTCGAGGCTCAAGAAGCCTCCTGTGTCA

**Drug**	**Vendor**	**Catalog Number**
Docetaxel	Selleckchem	S1148
Cabzitaxel	Selleckchem	S3022
VE-822	Selleckchem	S7102
Ribociclib	Selleckchem	S7440
Abiraterone	Selleckchem	S1123

Enzalutamide	Selleckchem	S1250

Supplementary Table 2: quantitative analysis of cell cycle profiles

LNCaP Wild Type

	G0 / G1 phase	S phase	G2/M phase
%Gated	66.7	9.0	22.9
Mean	1501.3	2103.6	2827.9

LNCaP LV-control + si-scramble

	G0 / G1 phase	S phase	G2/M phase
%Gated	64.9	13.4	18.2
Mean	1406.4	2102.6	2773.2

LNCaP o.e.MYBL2 + si-KDM5D

	G0 / G1 phase	S phase	G2/M phase
%Gated	64.5	12.2	20.1
Mean	1385.4	2150.3	2734.8

LAPC4 Wild Type

	G0 / G1 phase	S phase	G2/M phase
%Gated	58.5	14.7	25.5
Mean	1226.3	1757.5	2311.5

LAPC4 LV-control + si-scramble

	G0 / G1 phase	S phase	G2/M phase
%Gated	59.4	14.6	24.9
Mean	1252.3	1753.4	2303.1

LAPC4 o.e.KDM5D + si-MYBL2

	G0 / G1 phase	S phase	G2/M phase
%Gated	40.5	12.7	45.7
Mean	1150.1	1769.5	2242.7
